# Toward an Electronic Tongue Based on Surfactant-Stabilized Chemosensory Microparticles with a Dual Detection Mode

**DOI:** 10.1021/acsami.2c14800

**Published:** 2022-10-26

**Authors:** Aleksandra Kossakowska, Katarzyna Kociszewska, Kinga Kochman, Kamil Wojciechowski, Łukasz Górski, Patrycja Ciosek-Skibińska

**Affiliations:** Chair of Medical Biotechnology, Faculty of Chemistry, Warsaw University of Technology, Noakowskiego 3, 00-664Warsaw, Poland

**Keywords:** electronic tongue, microparticles, optodes, differential sensing, sensor array

## Abstract

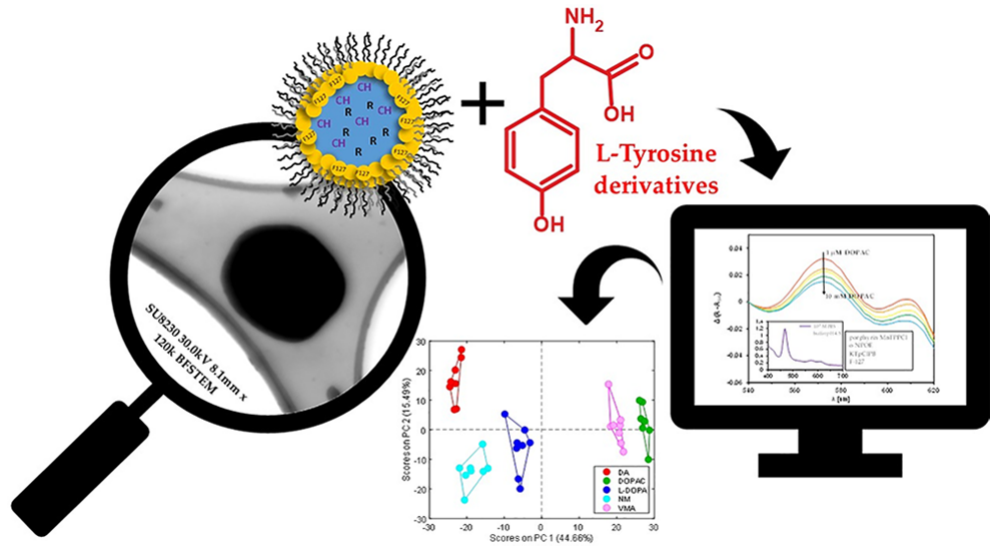

We propose a novel type of electronic tongue based on four types of monodispersed chemosensory microparticles (MPs) with a lipophilic core stabilized by a nonionic poloxamer surfactant. The lipophilic core composition was designed to achieve cross-sensitivity toward various ions and to enable spectrophotometric and/or spectrofluorimetric detection. Thus, generic anion-selective MPs, generic cation-selective MPs, as well as two types of metalloporphyrin-based MPs were fabricated and their morphology was characterized. Next, their differential sensing ability toward the discrimination of five l-tyrosine derivatives (dopamine, 3,4-dihydroxyphenylacetic acid, 3,4-dihydroxy-l-phenylalanine, normetanephrine, 4-hydroxy-3-methoxymandelic acid) was assessed. Comparison with the respective ion-selective electrode (ISE) responses was also provided to verify if the results from the potentiometric e-tongue correspond to outputs of the developed MP optode array. The recognition of dietary supplements containing l-tyrosine (l-Tyr) derivatives with the use of the MP-based e-tongue proved the potential of the developed sensing assay in pharmaceutical analysis.

## Introduction

Differential sensing strategy relies on recognition of a characteristic signal pattern, the so-called “fingerprint”. Such a process is typical for smell and taste recognition in mammals, where several “cross-sensitive” receptors provide a unique stimulus that can be stored in the brain and compared with new ones in the future. In chemistry, this strategy is adapted by the electronic nose and electronic tongue devices using cross-sensitive sensors or receptors.^1−3^ Their arrays provide signal patterns that are decoded for the extraction of analytically significant information. This offers an attractive alternative to the classical lock and key recognition because highly selective binding recognition sites are no longer essential for selective recognition. Moreover, various properties that are related to the sample characteristics can also be determined, such as the product origin, its aging time, or any real sample feature that is dependent on its many constituents. The differential sensing approach has proven utility in solving many complex analytical problems.^4−6^ The cross-sensitive sensors rely on some general features such as lipophilicity (e.g., generic anion-selective and cation-selective electrodes used in potentiometric electronic tongues^7−9^) or the ability to interact with specific groups of compounds, e.g., (metallo)porphyrins used as receptors in electronic noses and tongues.^10−12^

Metalloporphyrins are a class of very popular receptors for both potentiometric and optical sensors.^13^ Their usefulness as hosts is related to their stability, lipophilicity, optical properties, and the possibility to form porphyrin complexes with various metal cations. Interaction of anions with a metalloporphyrin takes place via their coordination as axial ligands to the central metal cation of the porphyrin structure. This results in the different selectivity observed for porphyrin complexes with various metal cations, making metalloporphyrins a very versatile group of receptors. The structure of the porphyrin core can also influence receptor selectivity to some extent; however, it mainly affects its optical properties. Possibly the most widespread is the use of metalloporphyrins as ionophores in polymeric membrane ion-selective electrodes.^14^ Porphyrin complexes with metal cations of +2, +3, and +4 charges were used for preparation of electrodes selective toward a wide array of anions.^15^ It is worth mentioning that the first metalloporphyrin used as the ionophore was Mn(III)[TPP]Cl (TPP = tetraphenylporphyrin).^16,17^ An elevated selectivity toward chloride and salicylate was reported for this ionophore, however, with selectivity coefficients only slightly better than for the electrodes based on an anion exchanger. The Mn(III) porphyrins were also proposed as ionophores for determination of I^-^ and SCN^–^ anions.^18,19^ Ion-selective electrodes (ISEs) based on metalloporphyrins were also used for determination of organic compounds, such as drugs diclofenac,^20^ 2-hydroxybenzhydroxamate,^21^ and sulfadiazine.^22^ Another interesting group of organic ions that can be detected with such electrodes constitute anions of carboxylic acids: salicylate,^23^ citrate,^24^ and acetate.^25^

Metalloporphyrins possess some unique optical parameters with very high molar extinction coefficients. This allowed the development of many sensors with the optical transduction mode. Ion-selective optodes can be fabricated with polymeric membranes of composition similar to those used for ion-selective electrodes. The change of a metalloporphyrin absorption/emission spectrum can be caused either directly by coordination of an analyte to the porphyrin metal center or by dissociating a dimeric form of the metalloporphyrin due to interaction with an analyte. One clear advantage of optical sensors over ion-selective electrodes is the possibility of detecting electroneutral substances. Therefore, metalloporphyrin-based sensors were developed for the determination of simple inorganic molecules, such as NO_2_^26^ and O_2_,^27^ as well as organic compounds, e.g., pyridine,^28^ biogenic diamines,^29^ or explosives.^30^

Both the ion-selective electrodes and ion-selective optodes can be miniaturized, which not only reduces consumption of chemicals used for the chemosensory-layer fabrication but also minimizes the sample volume. The latter is especially advantageous in electronic nose and electronic tongue applications, which require dozen-hundreds of samples for calibration. Recently, monodisperse ion-selective nano-/microspheres were proposed, containing a lipophilic pH indicator, an ion exchanger, a plasticizer, and (optionally) an ionophore.^31^ Their composition can be optimized to achieve the desired properties in terms of the selectivity pattern and particle size.^6,32,33^ Moreover, their pH cross-response and limited selectivity toward specific ions, which would be considered disadvantageous in classical sensing, are in fact advantageous in differential sensing.^6^

**Figure 1:**
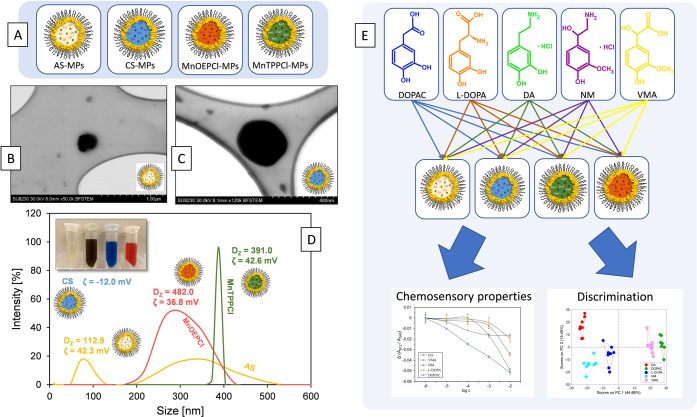
Pluronic-stabilized chemosensory microparticles as sensing units in MP optode-based electronic tongue: (a) schematic representation of four types of the studied MPs (AS-MPs, anion-selective microparticles; CS-MPs, cation-selective microparticles; MnOEPCl MPs and MnTPPCl MPs, microparticles incorporated with respective metalloporphyrins, details in Table 1). (b–c) STEM images of the fabricated AS-MPs and CS-MPs, respectively. (d) DLS results and zeta potentials of the developed MP optodes. (d) Study of MP chemosensory properties toward l-Tyr derivatives.

Therefore, in this work, we propose a novel type of electronic tongue based on chemosensory microparticles (MPs) with a lipophilic core stabilized by a nonionic poloxamer surfactant. The lipophilic core components reflect the composition of bulk ion-selective optodes with emphasis given to achieve cross-sensitivity. Thus, anion-selective (AS) and cation-selective (CS) microspheres, as well as two types of metalloporphyrin-based microspheres, were fabricated and characterized. Their differential sensing ability toward the discrimination of a series of structurally similar l-tyrosine derivatives (Figure 1) was investigated. Comparison with the respective ISE responses was also provided to verify if the results from the potentiometric e-tongue correspond to outputs of the proposed MP optode array. Finally, the application of the MP-based electronic tongue to the recognition of dietary supplements containing l-Tyr derivatives was shown to prove the suitability of the new approach in pharmaceutical analysis.

## Experimental Section

### Reagents and Materials

Dopamine hydrochloride (DA), 3,4-dihydroxyphenylacetic acid (DOPAC), 3,4-dihydroxy-l-phenylalanine (l-DOPA), DL-normetanephrine hydrochloride (NM), DL-4-hydroxy-3-methoxymandelic acid (VMA), sodium phosphate monohydrate, disodium phosphate dodecahydrate, HEPES, Tris, MES, Pluronic F127, and metalloprophyrins (2,3,7,8,12,13,17,18-octaethyl-21H,23H-porphine manganese(III) chloride—MnOEPCl; 5,10,15,20-tetraphenyl-21H,23H-porphine manganese(III) chloride—MnTPPCl) were supplied by Sigma-Merck (Poznań, Poland). Milli-Q water was used for the preparation of all aqueous solutions, including the phosphate buffers pH 3.0, 4.5, and 7.4. Plasticizers: 2-nitrophenyl octyl ether (*o*-NPOE), bis(2-Ethylhexyl) sebacate (DOS); lipophilic salts: tridodecylmethylammonium chloride (TDMAC), potassium tetrakis3,5-bis(trifluoromethyl)phenyl borate (KTFPB), sodium tetrakis[3,5-bis(trifluoromethyl)phenyl]borate (NaTFPB), potassium tetrakis(4-chlorophenyl)borate (KTpClPB); chromoionophores: chromoionophore I (9-(diethylamino)-5-(octadecanoylimino)-5H-benzo[*a*]phenoxazine, ETH 5294), chromoionophore XI (fluorescein octadecyl ester, ETH 7061); and poly(vinyl chloride), PVC, were obtained from Fluka (Selectophore). Real samples S1, S2, S3, S4, and S5 are dietary supplements containing l-DOPA, l-Tyr, or its derivative, produced by various manufacturers and purchased from various sources in Poland (supplement shops, pharmacies). Tetrahydrofuran (Fluka) was used as a solvent for the microsphere components. All chemicals were used as received.

### Preparation, Morphology, and Measurements of MP Optodes

Four types of optical MPs were prepared: sensitive to lipophilic anions (AS-MPs) and cations (CS-MPs) and two types of MPs based on metalloporphyrins: MnTPPCl and MnOEPCl (Table 1, Figure 1A). Their compositions were inspired by literature reports,^31^ modified according to our previous experience with AS and CS-ISEs employed in the electronic tongue sensor arrays^34−36^ and tested for differential sensing as AS/CS chemosensory optodes for the recognition of model bioanalytes.^6^ The cocktails of CS/AS microspheres consisted of the respective chromoionophore, a surfactant (Pluronic F127), a plasticizer, and the respective lipophilic salt playing the role of an ion exchanger. In the cases of MnTPPCl and MnOEPCl MPs, the chromoionophore was replaced by the respective metalloporphyrin serving both as a receptor and as an optical reporter. Each mixture was dissolved in 1.5 mL of tetrahydrofuran (THF) to obtain a homogeneous solution. To ensure complete dissolution of the mixture, each vial was placed in an ultrasonic bath (Sonic-0.5, 80 W, 40 kHz, POLSONIC Palczynski Sp. J., Warsaw, Poland). The next step was to pipette 0.5 mL aliquots into 4.5 mL of deionized water on a Vortex. The obtained mixture was blown with compressed air using a peristaltic pump. This treatment removes the organic solvents and provides a clear suspension of the particles.

**Table 1:** Compositions of MPs Forming an Array of Chemosensory Optode-Based Electronic Tongue

sphere ID	surfactant	plasticizer	ion exchanger	chromoionophore/Ionophore	optical properties
AS-MPs	F127, 12.5 mg	*o*-NPOE, 4 mg	TDMAC, 0.59 mg	ETH 7061, 0.6 mg	λ_max_ = 454 nm λ_ex_/λ_em_: 463 nm/555 nm
CS-MPs	F127, 12.5 mg	DOS, 4 mg	KTFPB, 1.55 mg	ETH 5294, 0.5 mg	λ_max_ = 610 nm λ_ex_/λ_em_: 614 nm/686 nm
MnOEPCl MPs	F127, 12.5 mg	*o*-NPOE, 4 mg	KTpClPB, 0.48 mg	MnOEPCl, 2 mg	λ_max_ = 456 nm
MnTPPCl MPs	F127, 12.5 mg	*o*-NPOE, 4 mg	KTpClPB, 0.425 mg	MnTTPCl, 2 mg	λ_max_ = 468 nm

For all measurements, freshly prepared MPs were used. Their size was confirmed using a scanning transmission microscope using transmission measurements (STEM, Hitachi SU8230 FE SEM). The particle size distributions and ζ-potential values were determined at 25 °C using a Malvern Zetasizer 3000HS equipped with a 633 nm laser. The dynamic light scattering (DLS) results are presented as scattering intensity-based size distributions and *z*-average particle diameters (*D*_z_).

All spectrophotometric and spectrofluorimetric measurements were performed in at least four replications by a Synergy MX Multi-Mode microplate reader (BioTek Instruments, Inc., Winooski, VT) using 96-well Greiner CELLSTAR polystyrene microplates (Greiner Bio-One GmbH, Kremsmünster, Austria). The MP suspensions in a microtiter well were obtained by pipetting 100 μL of AS-MPs or CS-MPs or 25 μL of MnTPPCl MPs with 75 μL of deionized water, or 50 μL of MnOEPCl MPs with 50 μL of deionized water. Then, 100 μl of buffered (0.01 M PBS pH 3.0/4.5/7.4) analyte solution in the concentration range 10^–6^–10^–1^ M was added.

For the discrimination analysis, independent measurements were made in eight replications. In this case, the analytes, l-tyrosine derivatives (Figure 1E), at a concentration of 1 mM were tested in buffered conditions (0.01 M HEPES pH 7.4 for AS-MPs, 0.01 M Tris-HCl pH 9 for CS-MPs, 0.01 M MES pH 4.5 for the metalloporphyrin-based MPs). Preparation of dietary supplements consisted of dissolving a dose (1 tablet) in water up to 25 mL and placing in an ultrasonic bath for 20 min to facilitate the release of API and all excipients. Finally, the resulting solution was filtered by a syringe filter (PTFE, 0.2 μm) and then spotted to microwells of the microtiter plate containing the respective MP suspensions.

### Preparation and Measurements of ISEs

The compositions of all membranes used for potentiometric measurements are shown in Table 2. All membrane components were dissolved in 2 mL of THF, and the mixture was cast in a 24-mm-i.d. glass ring mounted on a glass slide. The solvent was allowed to evaporate overnight. Seven-mm-diameter discs were cut out from this parent membrane and mounted in IS-561 electrodes. Electrochemical potentials of these electrodes were measured against a double-junction Ag/AgCl reference electrode using an EMF-16 Lawson Labs data acquisition system. Phosphate-buffered solutions of pH 3.0, 4.5, and 7.4 were employed for preparation of sample solutions. Aliquots of 0.01 M solutions of the tested substances were added to 50 mL of stirred buffer to perform calibration of the tested electrodes.

**Table 2:** Compositions of Membranes of ISEs Forming an Array of Potentiometric Electronic Tongue

ISE ID	polymer	plasticizer	ionophore	ion exchanger
AS-ISE	PVC, 66 mg	*o*-NPOE, 132 mg		TDMAC, 2 mg
CS-ISE	PVC, 66 mg	DOS, 132 mg		NaTFPB, 2 mg
MnOEPCl-ISE	PVC, 66 mg	*o*-NPOE, 132 mg	MnOEPCl, 2 mg	KTpClPB,0.425 mg
MnTPPCl-ISE	PVC, 66 mg	*o*-NPOE, 132 mg	MnTPPCl, 2 mg	KTpClPB, 0.425 mg

### Data Analysis

The data acquired for the MP optodes were obtained in a form of UV–vis absorption spectra and fluorescence emission curves. Values of absorbances and fluorescence intensities in the respective maxima of peaks of four types of fabricated microspheres were used according to Table 1. For the classification analysis of l-Tyr derivatives, the entire spectra were used as fingerprints for specific bioanalytes. The differential sensing ability was verified using an unsupervised chemometric technique—principal component analysis (PCA). Before the chemometric analysis, preprocessing was applied: mean centering in the case of optodes and autoscaling in the case of potentiometric results. All data analyses were performed using Solo software (Eigenvector Research Inc., Manson, WA), while the calibration plots were generated in MS Excel 2020 (Microsoft, Redmond, WA) and Origin.

## Results and Discussions

### Mechanisms of Optical Signal Generation and Morphology of MP Optodes

The ISE membranes and chemosensory MPs were prepared according to the experimental section’s guidelines and Tables 1 and 2, giving emphasis to the similarity in composition of the respective sensor types. In the past, we developed and employed the AS-ISEs and CS-ISEs for electronic tongue sensor arrays serving in a wide range of applications,^34−39^ whereas the metalloporphyrin-based ISEs were developed in our group for selective sensing of fluoride, acetate ions, as well as the total concentration of aliphatic carboxylic acids.^25,40−42^ Accordingly, the composition of four types of chemosensory microspheres (Table 1) was adjusted to obtain similar functionality to their ISE counterparts but different transduction of the signal. In our previous work, we showed that such chemosensory nano-/microspheres can be fabricated reliably with a high degree of repeatability, and their lifetime exceeds 2 months.^6^

The microspheres selective for both anions (A) and cations (C) contained a chromoionophore (CH) as an optical transducer, which can be protonated/deprotonated in the presence of an ion exchanger (R). This change in the protonation degree of the chromoionophore is responsible for the change in the optical properties of the produced microspheres according to eqs 1 and 2 (for AS-MPs and CS-MPs, respectively).^6,32,33^ The next two types of microspheres were incorporated with the manganese porphyrins, which played the double role of an ionophore and an optical transducer, according to eqs 3 and 4.
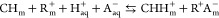
1
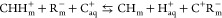
2
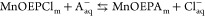
3
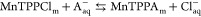
4

The microspheres were produced by precipitation, which is much less demanding in terms of time and labor as compared to other methods, like sonication or polymerization. It allows for obtaining the micrometric particle size in a simple process.^31^Figure 1B,C shows exemplary images obtained using a scanning transmission electron microscope (STEM) with ultrahigh resolution. The carbon mesh was immersed in the MP suspension and left for solvent evaporation. The MPs were observed using a bright field detector (BF-STEM) to obtain the best possible image. The observed sizes of the pluronic-stabilized spheres were in the range of 50–400 nm. Their size depends on many factors such as mixing speed during fabrication, the type and concentration of components, etc., which were confirmed in our previous studies.^32,33^ Although STEM allowed for direct imaging of the microparticles, confirming their spherical shape, the STEM sample preparation (solvent evaporation) may affect the observed size of the spheres due to aggregation. To confirm the presence of nano-/microspheres in the undiluted dispersion of the MP optodes, the particle size distribution was analyzed using dynamic light scattering (DLS, Figure 1D). Except for CS, the *z*-average diameter (*D*_z_) for all MPs did not exceed 400 nm, which is in agreement with the STEM analysis. This suggests that the particles do not undergo significant aggregation upon drying, which is at least partly due to their high absolute ζ-potential values (see below). Unfortunately, the DLS size measurements of CS-MPs could not be completed due to the strong light absorption of the employed chromoionophore (λ_max_ = 612 nm) overlapping with the wavelength of the laser used in DLS (633 nm). The most monodisperse MPs were observed for MnTPPCl, while a clearly broader distribution was observed for MnOEPCl. Although the two MPs share practically the same matrix (polymeric surfactant, plasticizer, ion exchanger), they differ in the chemical structure of the porphyrin, which may apparently affect the particle distribution. Nevertheless, the *z*-average diameters are very close for both porphyrin-based MPs, and the observed differences in size distribution might simply reflect slight differences in their preparation protocol. On the other hand, the AS-MPs show a bimodal distribution with significantly lower *D*_z_, which may stem from a different plasticizer employed in their composition (DOS instead of o-NPOE).

The ion-selective potential of all four investigated MPs is evidenced by the sign of their ζ-potential: all anionic-selective compositions display positive values, while CS-MPs show a clearly negative ζ-potential value. Given the nonionic character of the polymeric surfactant, plasticizer, and chromoionophores, the microspheres’ surface charge is governed by the metalloporphyrin and the ion exchanger. Both porphyrin-based MPs display a clearly positive surface potential, reflected in ζ-potential values around +40 mV, despite the presence of the ion exchanger with a large, potentially surface-active, tetrakis(4-chlorophenyl)borate anion. This suggests that the particles’ surface is enriched with the positively charged metalloporphyrin, rather than with the ion exchanger. The opposite is observed for CS-MPs, where the chromoionophore is nonionic and the slightly negative surface charge (and consequently ζ-potential) originates only from the tetrakis[3,5-bis(trifluoromethyl)phenyl]borate anion. Interestingly, the tridodecylmethylammonium cation-based ion exchanger in the anion-selective MPs provides a much higher absolute value of the surface potential (but with the opposite sign). This is reflected in the highly positive ζ-potential value for AS-MPs, comparable to that of both metalloporphyrin-based MPs. The surface accumulation of tetraalkylammonium cations has been shown previously to give rise to the anionic selectivity of air–water^43^ and ISE–water^44^ interfaces. Besides the ion selectivity, the relatively high ζ-potential (in terms of absolute values) probably contributes also to the long-term stability of the MP dispersions during operation (at least 8 weeks) and negligible aggregation during sample drying for STEM preparation. Even for CS-MPs, which exhibited a lower (in absolute terms) ζ-potential, no significant aggregation could be noticed in STEM.

### Chemosensory Properties of the Developed MPs

**Figure 2:**
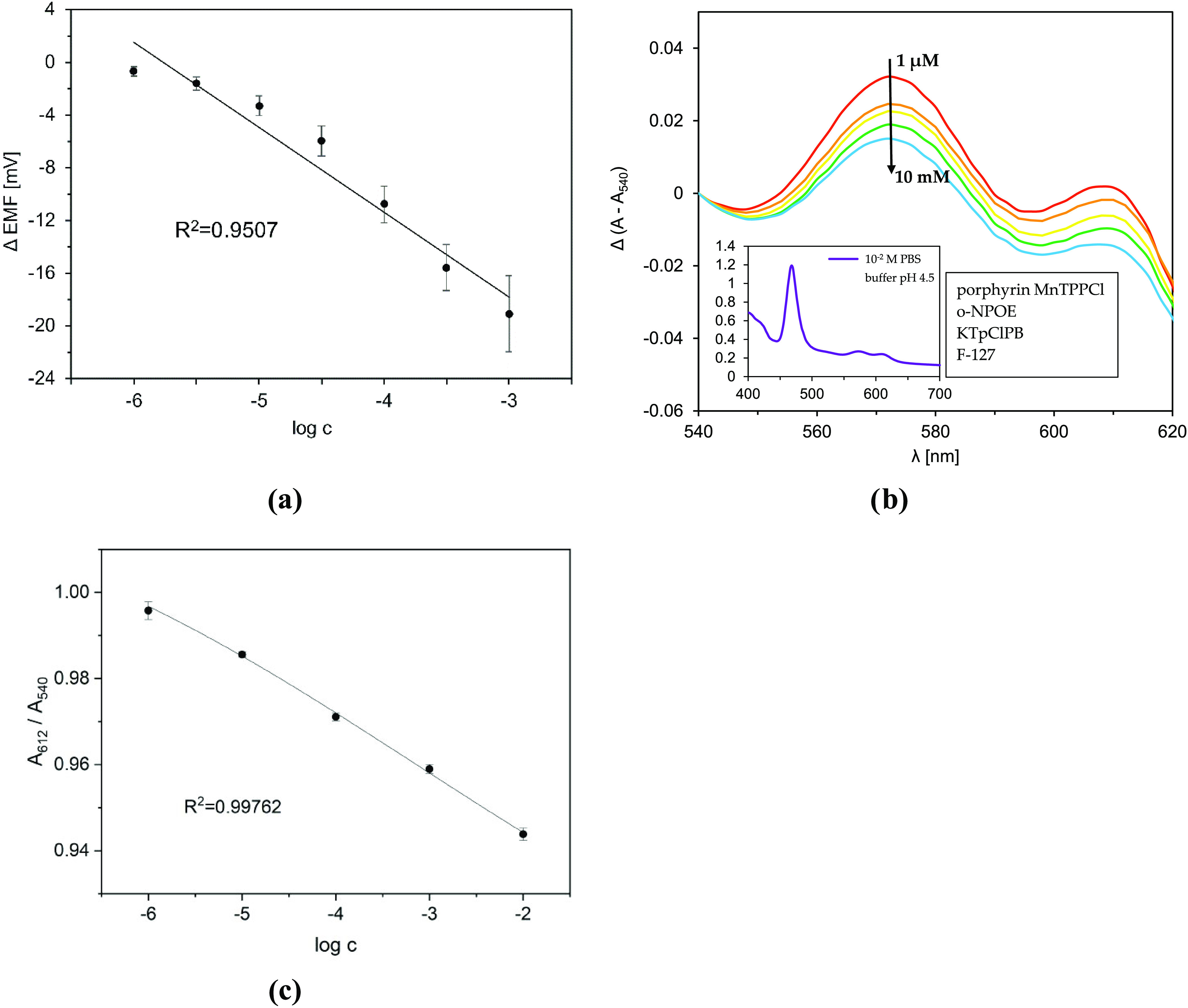
Potentiometric (a) and spectrophotometric (b, c) responses of sensors based on the MnTPPCl porphyrin toward DOPAC: (a) calibration curve of MnTPPCl-ISE; (b) UV–vis spectrum of MnTPPCl MPs in the sensing range (entire UV–vis absorption spectrum in the inset); and (c) respective calibration curve. Each solution of DOPAC was buffered (0.01 M phosphate buffer, pH 4.5 for spectrophotometric measurements and 0.05 M phosphate buffer, pH 4.5 for potentiometric measurements). Points of the calibration curve were determined as mean ± SD, *n* ∈ {3,4}.

First, the response of MnTPPCl-based sensors toward one l-Tyr derivative (DOPAC) was analyzed. As expected, an anionic response of MnTPPCl-ISE was recorded, most likely due to the presence of a carboxylic group in the DOPAC structure (Figure 2a). However, the response was rather weak, with a significantly sub-Nernstian slope of −9 mV dec^–1^ and a linear response in the DOPAC concentration range from 10^–5^ to 10^–3^ M. In the case of MnTPPCl MPs, the characteristic metalloporphyrin peaks were observed in the UV–vis spectrum (Figure 2B, inset), with a Soret band at 468 nm and two peaks in the Q-band region at 572 and 608 nm.

The absorbance in the Q-band region (540–620 nm, Figure 2b) was changing linearly with the analyte concentration; hence, this part of the spectrum was used for construction of the calibration curve (Figure 2c). The linear range spanned the whole range of the employed concentrations (1 μM to 10 mM). Thus, it can be concluded that chemosensory responses of both MnTPPCl-based sensors are comparable, and MnTPPCl MPs can provide an even lower limit of detection than their ISE counterpart. Very small error bars for the former sensors prove the high repeatability of the measurements performed with the developed MPs.

**Figure 3:**
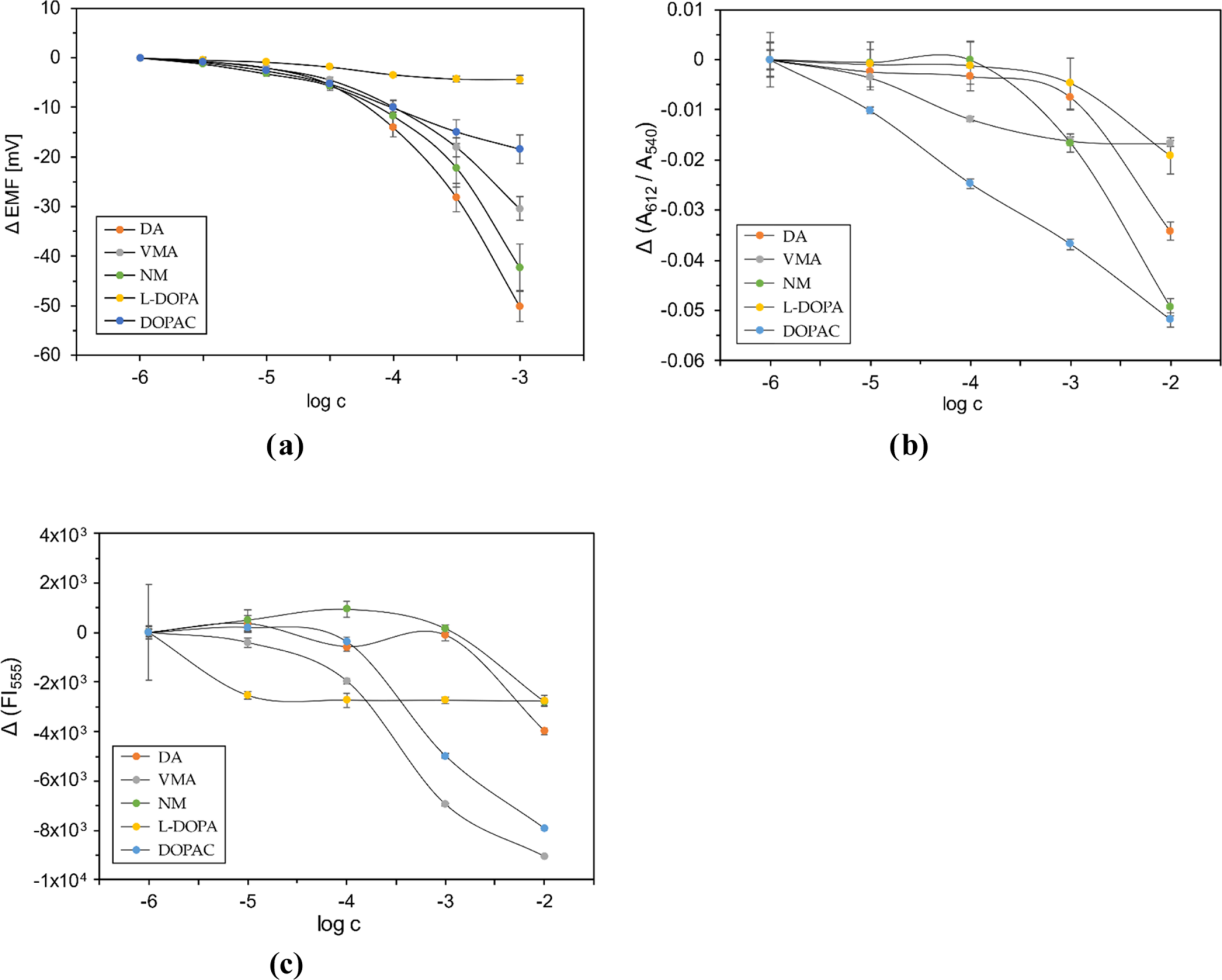
Exemplary potentiometric (a), spectrophotometric (b), and spectrofluorimetric (c) responses of the fabricated sensors toward l-tyrosine derivatives: (a) MnTPPCl-ISE; (b) MnTPPCl MPs (signal based on the change in the ratio of absorbances in 612 and 540 nm); (c) AS-MPs (signal based on the change of FI for λ_ex_/λ_em_ 463 nm/555 nm). All solutions were buffered (0.01 M phosphate buffer pH 4.5 for spectrophotometric measurements and 0.05 M phosphate buffer, pH 4.5 for potentiometric measurement). Points of calibration curves were determined as mean ± SD; *n* ∈ {3,4}.

The next series of experiments was dedicated to testing the sensitivity of the developed sensors toward various l-Tyr derivatives. All five analytes, DOPAC, DA, VMA, l-DOPA, and NM, were employed to calibrate both ISEs and MPs. Some representative responses are provided in Figure 3. The highest sensitivity for MnTPPCl-ISE was observed for DA and NM, followed by VMA and DOPAC (Figure 3a). The latter show superior selectivity toward chloride anions over the carboxylic group, which is consistent with the literature data.^19,45^ The lowest response was noticed for l-DOPA, which is not surprising given its highest hydrophilicity (log *P* = −2.39).

In the case of MnTPPCl MPs, significant responses were observed also for DA and NM, followed by VMA. However, in this case, the highest absorbance change was noted for DOPAC (Figure 3b). It evidences that the response of chemosensory MPs is governed mainly by the lipophilicity of the target analyte. Thus, DOPAC, the most hydrophobic compound in the studied set (log *P* = +0.98), exhibits not only the highest sensitivity but also the widest linear range. It should also be noticed that the limit of detection for all studied compounds is at the same level for ISEs (ca 10 μM), while for MPs it can be lower (DOPAC), the same (for VMA), or higher (DA, NM), resulting in different ranges of linear response. Thus, the sensitivity patterns of ISEs and MPs bear some similarities, but also significant differences are evident, making them rather complementary than duplicating types of sensors. The differences stem mainly from the fact that potentiometric sensors are selective only toward ions, whereas optical sensors can also respond toward neutral analytes.

The dominant role of lipophilicity of the target ion in the MP responses was also visible in the fluorescence detection mode of AS-MPs (Figure 3c), which contain only an ion exchanger. As expected, the highest sensitivity was observed for anions with log *P* > 0: DOPAC, with log *P* = +0.98, and VMA, with log *P* = +0.43 (note that the pH of the employed buffer was higher than the p*K*_a_ values for both DOPAC and VMA). DA and NM, which are chlorides of amines under the employed measurement conditions, showed similar responses to each other, lower than for DOPAC and VMA. The most hydrophilic l-DOPA anions (log *P* = −2.39) influenced the MP fluorescence response only slightly. Therefore, the selectivity pattern of AS-MPs is consistent with the ion-exchange mechanism, confirming their proper design and suitability for differential sensing.

### Selectivity Patterns of ISE and MP Optodes toward l-Tyr Derivatives

**Figure 4:**
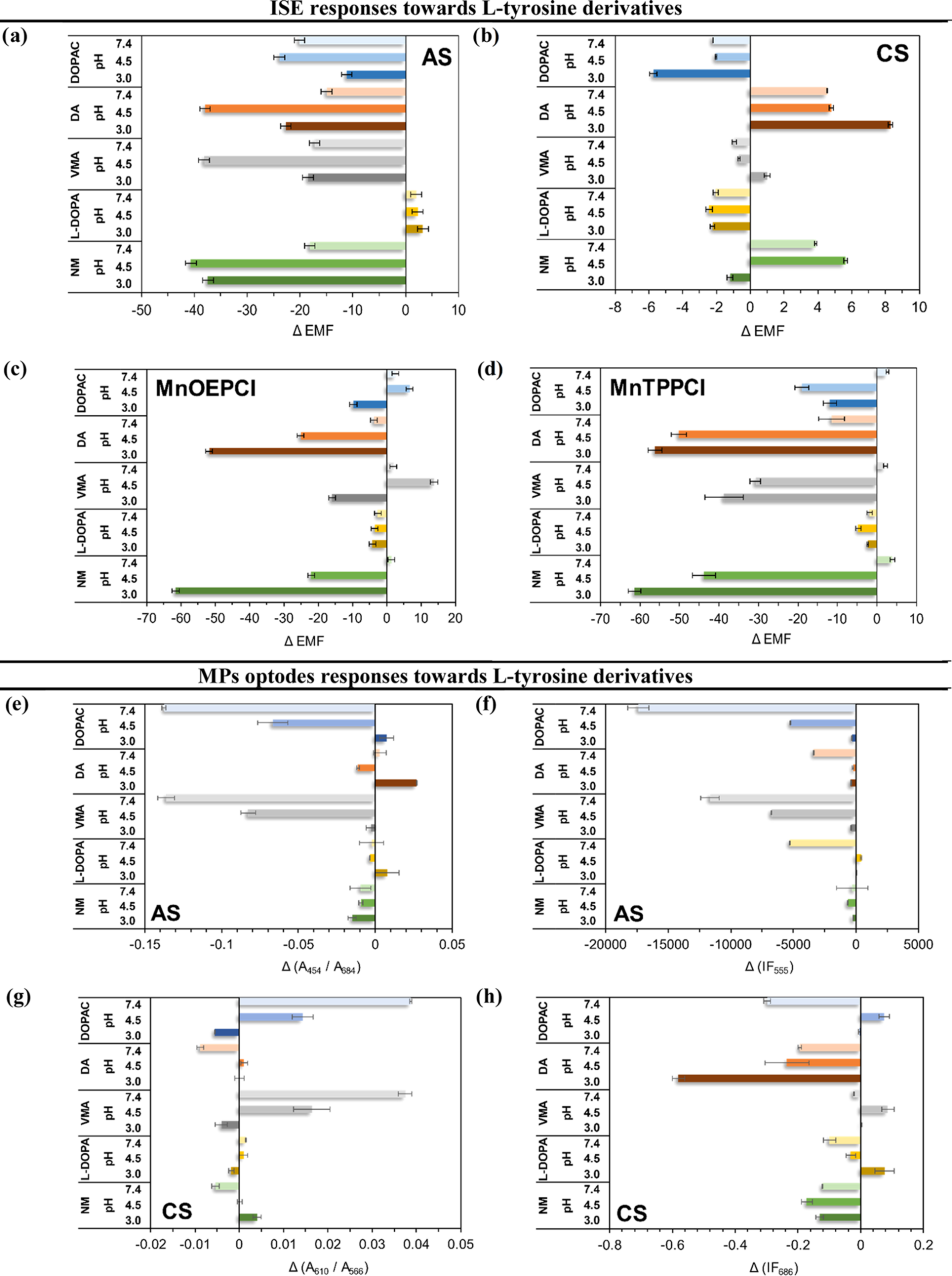

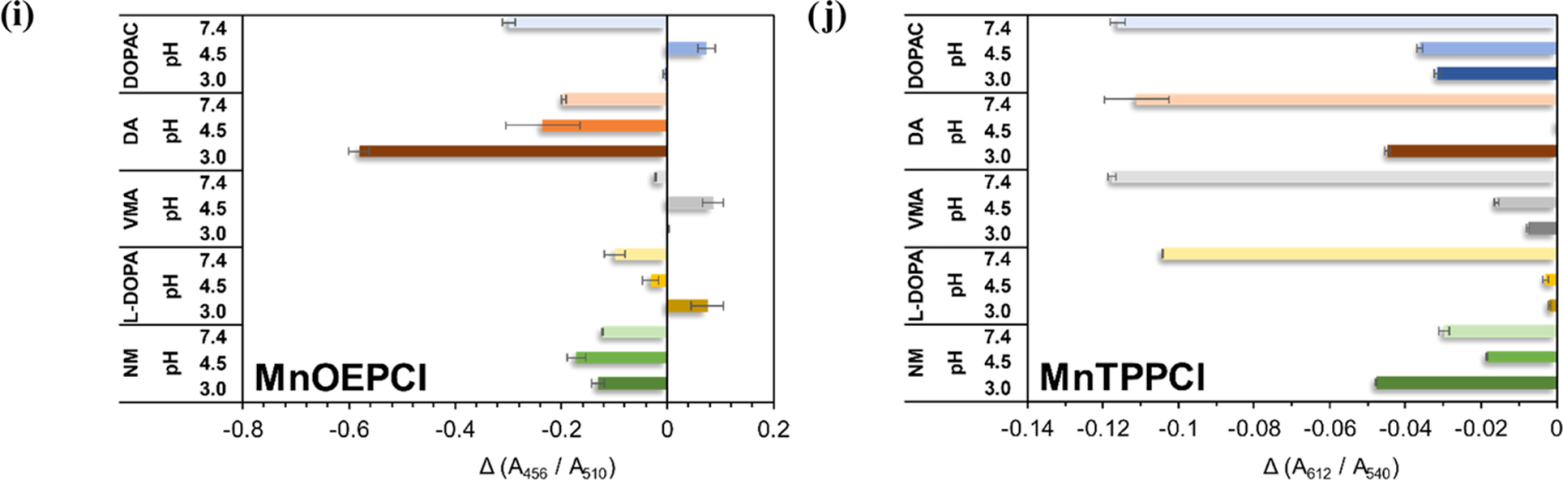
Potentiometric (a–d), spectrophotometric (e, g, i, j), and spectrofluorimetric (f, h) responses of the fabricated sensors toward l-tyrosine derivatives at the 1 mM concentration level (mean ± SEM; *n*∈ {3,4}) at varying pHs. ΔEMF is the difference between the EMF obtained in the buffered analyte solution and in pure 0.05 M phosphate buffer of the respective pH. Δ(*A*_max_/*A*_base_) is the difference between the ratios of respective absorbances (Table 1) obtained in the buffered analyte solution and in pure 0.05 M phosphate buffer of the respective pH. Δ(IF_max_) is the difference between fluorescence intensities at peak maximas (Table 1) obtained in the buffered analyte solution and in pure 0.05 M phosphate buffer of the respective pH.

Having confirmed the existence of varied response patterns of ISEs and MPs toward various l-Tyr derivatives, which are the prerequisites for differential sensing, we extended the study to all remaining sensors. As pH affects the analytes’ chemical form, as well as the receptor layer preferences, ion-exchange processes, and the starting level of the protonation degree of the chromoionophores, all calibrations (concentration range 1 μM to 1.0 mM) were performed at three pH levels, for all derivatives and sensors. The results are presented as the respective signal changes in Figure 4. The first general observation is that the selectivity patterns indeed differ from each other (are not redundant), although in some cases, some similarities can be found. Second, ISEs and MPs are not exact counterparts, in agreement with observations in the section Preparation, Morphology, and Measurements of MP Optodes. The third remark is that both of the response magnitudes and the selectivity patterns are pH-dependent. Thus, pH alteration could be an efficient signal enrichment strategy for future applications of ISE and MP sensor arrays, which confirms also our previous findings.^6^ The signal enrichment can be alternatively achieved by combining the spectrophotometric and spectrofluorimetric modes of signal acquisition, as their outputs are not perfectly correlated (e.g., dopamine sensing, Figure 4g,h).

In agreement with our previous studies,^32,33^ AS-ISE and CS-ISE displayed generic anion- and cation selectivities, respectively (Figure 4a,b). The highest anionic responses of AS-ISE were observed at pH 4.5 and were comparable for DA, VMA, and NM. The lowest response noted for l-DOPA can be explained by its hydrophilicity (the lowest log *P* in the studied analyte set). The optical counterpart (AS-MPs) responded mostly to VMA and DOPAC, again confirming that the MP response is governed mostly by lipophilicity (only for these two analytes log *P* > 0), in contrast to their ISE analogues. Moreover, the AS optodes perform the best at a higher pH (pH 7.4, Figure 4e,f) than AS-ISE. This effect can be linked with a more complex signal transduction in MPs, involving an additional equilibrium-protonation of the chromoionophore. Not surprisingly, the cationic responses of CS-ISE (Figure 4b) were the most significant for DA and NM, both possessing an ionized amine group under the employed experimental conditions. Significant changes in the fluorescence signal were also observed for CS-MPs in the presence of DA and NM for all pH levels (Figure 4h); however, the DA response was more pronounced in this case. Such a selectivity pattern can be again explained by differences in the analyte lipophilicity (slightly higher in the case of DA), affecting the CS-MPs’ response (Figure 4h) to a higher extent than that of CS-ISE (Figure 4b).

The presence of ionophores in the ISE membranes or the MP cores may significantly alternate the selectivity pattern, as indeed observed for all sensors containing Mn porphyrins (Figure 4c,d,i,j). For MnOEPCl and MnTPPCl-based electrodes, selectivity clearly differs from the generic pattern of the corresponding AS-ISE (Figure 4c,d). Their sensitivity was the highest at pH 3.0, which can be explained by a competition between the analyte and OH- anions for the binding site of the ionophore’s metallic center.^40^ The affinity of both ionophores to the carboxylate and chloride anions is clearly reflected in the strong responses observed for DA, VMA, NM, and DOPAC.

Contrary to its ISE counterpart, MnTPPCl MPs presented the highest response at pH 7.4. MnTPPCl MPs are less selective, showing more generic response toward all derivatives studied (high and comparable response toward DOPAC, DA, VMA, and l-DOPA, Figure 4j) than MnOEPCl MPs, which show a preference for DA at pH 3.0 (Figure 4i). This corresponds well with observations of their ISE analogues (MnTPPCl-ISE and MnOEPCl-ISE, respectively).

To conclude, all sensors employed in the present study provide unique signal patterns, which can be exploited for differential sensing. Moreover, the ISE-based and MPs-based sensor arrays exhibit varied signal patterns toward the l-Tyr derivatives, which is a prerequisite for the electronic tongue recognition strategy.

### Discrimination of l-Tyr Derivatives and Dietary Supplements Based on Differential Sensing

In our previous work,^6^ we applied a chemosensory optode array based on pluronic-stabilized MPs for differential sensing. Its suitability for pattern-based identification was proved using a library of eight model bioanalytes belonging to the group of neurotransmitters: dopamine, epinephrine, norepinephrine, γ-aminobutyric acid (GABA), acetylcholine, histamine, taurine, and phenylethylamine. Despite similar biofunctionalities, they differed significantly in terms of the chemical structure. In this work, we attempted to apply the differential sensing ability of the developed MPs in a more complicated case of structurally similar derivatives of l-tyrosine. They all feature an aromatic ring with a hydroxyl group in the para position and a hydroxyl/methoxy group in the meta position; three of the five derivatives possess an additional amino group and/or a carboxyl group (Figure 1E).

The pH-buffered 1 mM solutions of all five analytes were subjected to the analysis with two types of electronic tongues:

- potentiometric electronic tongue formed by an array of four types of ISEs (AS-ISE, CS-ISE, MnOEPCl-ISE, MnTPPCl-ISE), and
- microparticle-based electronic tongue with assays combining four types of MPs (AS-MPs, CS-MPs, MnOEPCl MPs, MnTPPCl MPs).

**Figure 5:**
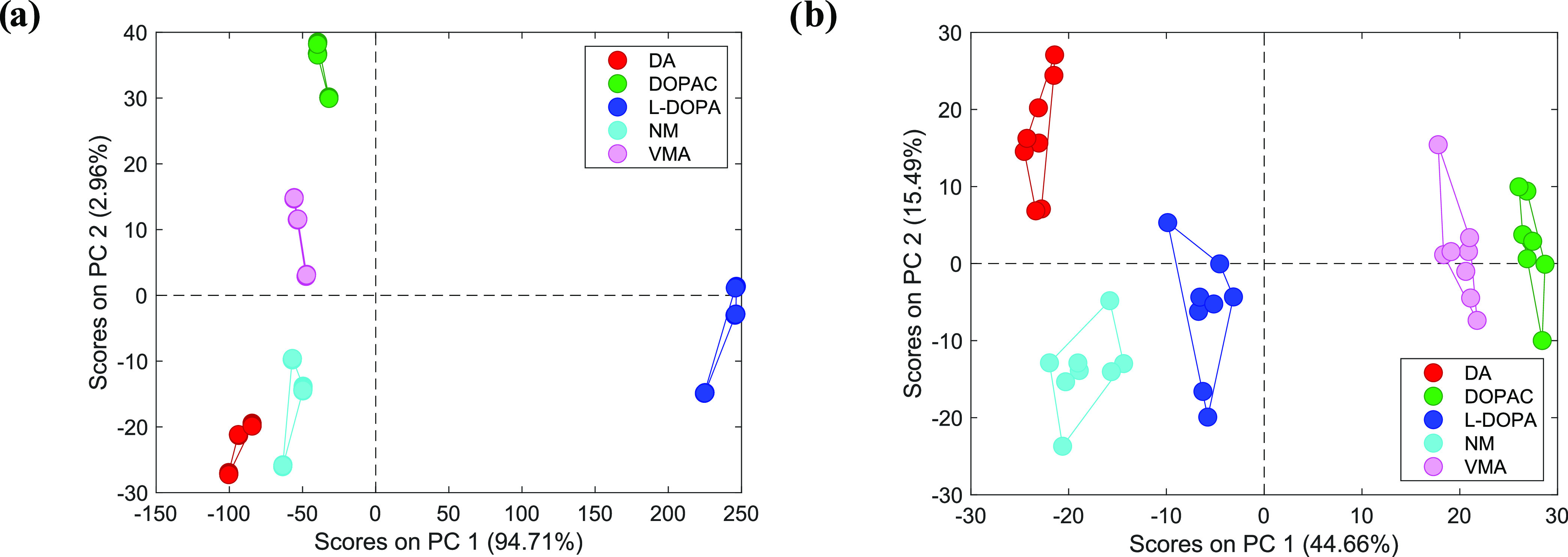
PCA score plots showing the discrimination of l-tyrosine derivatives at the 1 mM concentration level obtained by the (a) potentiometric electronic tongue and (b) MP optode-based electronic tongue with dual detection.

The recorded signals were compiled into two large, collective matrices, separately for the potentiometric and optical measurements. After preprocessing, they were subjected to a principal component analysis (PCA), as the most straightforward and unsupervised technique allowing visualization of the similarity of the studied samples characterized by the collected data. The results are presented as PCA score plots in Figure 5. First of all, it should be noticed that all five l-Tyr derivatives were easily discernable by both types of electronic tongues since separate clusters can be distinguished in the PC1–PC2 spaces. Interestingly, the cluster arrangements are quite similar for both sensing systems. DOPAC and VMA exhibit similar potentiometric and MP-based fingerprints. This can be justified by their close structural similarity: both derivatives feature the same main functional group (carboxyl) and both belong to the most lipophilic ones (log *P* > 0). Another pair exhibiting very close PC1 values for both sensing systems is DA and NM. Again, both molecules share the same structural element, the amine group, and display very similar lipophilicity (log *P* ≈ −1). On the other hand, the location of the l-DOPA cluster seems to be different on both plots of Figure 5; however, upon closer inspection, a similar arrangement can be noticed (yet on the other principal component). In the case of the MP-based electronic tongue, an increase of PC1 indicates discrimination between the amines (DA, NM), through l-DOPA (amino acid), to the carboxylic acids (DOPAC and VMA). Exactly the same arrangement is seen for the potentiometric electronic tongue, but along the PC2 axis: its increasing value shows transition from the amines, through the amino acid, to the carboxylic acids. It can be concluded that both the MP- and ISE-based electronic tongues offer a similar classification ability, yet they should not be regarded as exact substitutes. Anyway, the present results prove the great potential for the recognition of subtle differences in the chemical structure by employing differential interaction of microspheres with analytes.

**Figure 6:**
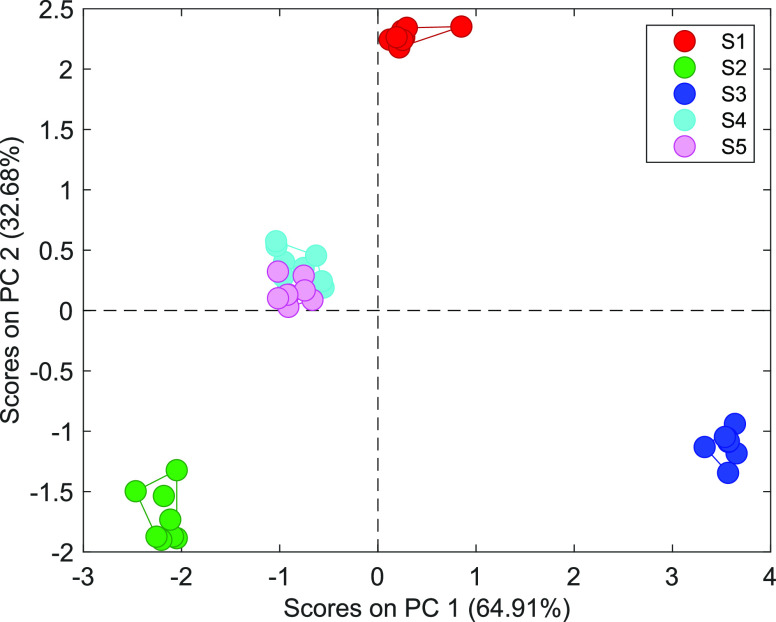
PCA score plot showing the discrimination of five dietary supplements by the MP optode-based electronic tongue with dual detection (S1: *N*-acetyl-l-tyrosine supplement; S2: l-tyrosine supplement; S3, S4, S5: l-DOPA supplements). The presented results were obtained using values of absorbances and fluorescence intensities in their respective peak maxima of four types of fabricated MPs (according to Table 1). S4 and S5 are supplements with the same composition but available under different brand names.

The last part of the research was dedicated to the use of the developed MP-based electronic tongue in a typical electronic tongue application: recognition of pharmaceuticals. l-Tyr and its derivatives are often employed as dietary supplements. l-Tyr is one of the most important amino acids for the human body. Its deficiency may contribute to disturbances in functioning of the thyroid gland and the development of depression, it may also cause fatigue and weakness. As l-Tyr is believed to have slimming, fatigue-reducing, and muscle-strengthening properties, it is increasingly used regularly by training people. In dietary supplements, it is present either in the free form or as its *N*-acetyl derivative, which facilitates its bioavailability due to increased solubility. l-DOPA, another popular supplementation in amino acid, is a direct dopamine precursor and an intermediate metabolite in the adrenaline synthesis pathway, thus having a great influence on an organism’s hormonal balance. Five commercially available dietary supplements based on l-Tyr or its derivatives were analyzed by the MP-based electronic tongue. Its spectrophotometric and spectrofluorimetric signals were processed by PCA and visualized on a PCA score plot in Figure 6. Most of the clusters are easily discernable from each other. The supplements containing l-Tyr or its *N*-acetyl derivative (S1, S2) can be easily identified, along with l-DOPA supplements (S3, S4, S5). The high overlap of clusters S4 and S5 proves the practical utility of the MP-based electronic tongue in pharmaceutical identifications because these two l-DOPA supplements have the same composition (i.e., both API and excipients are the same) but are available under different brand names for marketing reasons. It evidences that the MP-based electronic tongue with the dual detection mode possesses a high potential to supplement the research carried out with electronic tongues based on various measurement principles and used in pharmaceutical applications.

## Conclusions

In this work, we developed new chemosensory microparticles with the lipophilic core stabilized by the pluronic surfactant and employed them in the ion-sensitive optode array used for electronic tongue sensing. The set of four types of microsized cross-sensitive sensors could recognize subtle differences in the chemical structure of five l-Tyr derivatives, relying on the differential interaction of MPs with various moieties present in the studied molecules. It was shown that the microspheres can respond in the dual mode of detection (spectrophotometric and fluorimetric), which can help enhance the identification accuracy. Their optical response is also pH-dependent; thus, modulation of pH can be a simple and efficient signal enrichment strategy for their future applications. Fabrication of MPs is simple, versatile, and convenient, and it could be adopted to various quantitative and qualitative analytical tasks by simply adjusting the MP components and measurement conditions. They can be designed analogously to the ion-sensitive membranes of ISEs and bulk ion-sensitive optodes. However, as has been shown in this paper, the MP optodes are not exact substitutes of their ISE/bulk optode counterparts mainly because of their higher selectivity toward lipophilic analytes. Nevertheless, their differential sensing capabilities, as shown in an example of dietary supplement identification, make them promising candidates for future electronic tongue assays.
